# Insulin resistance and systemic metabolic changes in oral glucose tolerance test in 5340 individuals: an interventional study

**DOI:** 10.1186/s12916-019-1440-4

**Published:** 2019-11-29

**Authors:** Qin Wang, Jari Jokelainen, Juha Auvinen, Katri Puukka, Sirkka Keinänen-Kiukaanniemi, Marjo-Riitta Järvelin, Johannes Kettunen, Ville-Petteri Mäkinen, Mika Ala-Korpela

**Affiliations:** 1 Computational Medicine, Faculty of Medicine, University of Oulu, Oulu, Finland; 2 Systems Epidemiology, Baker Heart and Diabetes Institute, Melbourne, VIC Australia; 3 Center for Life Course Health Research, Faculty of Medicine, University of Oulu, Oulu, Finland; 4 Biocenter Oulu, University of Oulu, Oulu, Finland; 5 Unit of Primary Care and Medical Research Center, Oulu University Hospital, Oulu, Finland; 6 Oulunkaari Health Center, Ii, Finland; 7 NordLab Oulu, Oulu University Hospital and Department of Clinical Chemistry, University of Oulu, Oulu, Finland; 8 Health and Wellfare Center, Oulu, Finland; 9 Healthcare and Social Services of Selänne, Pyhäjärvi, Finland; 10 Department of Epidemiology and Biostatistics, MRC-PHE Centre for Environment and Health, School of Public Health, Imperial College London, London, UK; 11 Department of Life Sciences, College of Health and Life Sciences, Brunel University London, Uxbridge, Middlesex UK; 12 National Institute for Health and Welfare, Helsinki, Finland; 13 Computational and Systems Biology Program, Precision Medicine Theme, South Australian Health and Medical Research Institute, Adelaide, Australia; 14 Hopwood Centre for Neurobiology, Lifelong Health Theme, SAHMRI, Adelaide, Australia; 15 MRC Integrative Epidemiology Unit, University of Bristol, Bristol, UK; 16 Population Health Science, Bristol Medical School, University of Bristol, Bristol, UK; 17 NMR Metabolomics Laboratory, School of Pharmacy, University of Eastern Finland, Kuopio, Finland; 18 Department of Epidemiology and Preventive Medicine, School of Public Health and Preventive Medicine, Faculty of Medicine, Nursing and Health Sciences, The Alfred Hospital, Monash University, Melbourne, VIC Australia

**Keywords:** Insulin resistance, Metabolic profiling, Oral glucose tolerance test, Impaired glucose tolerance, Impaired fasting glucose, Type 2 diabetes

## Abstract

**Background:**

Insulin resistance (IR) is predictive for type 2 diabetes and associated with various metabolic abnormalities in fasting conditions. However, limited data are available on how IR affects metabolic responses in a non-fasting setting, yet this is the state people are mostly exposed to during waking hours in the modern society. Here, we aim to comprehensively characterise the metabolic changes in response to an oral glucose test (OGTT) and assess the associations of these changes with IR.

**Methods:**

Blood samples were obtained at 0 (fasting baseline, right before glucose ingestion), 30, 60, and 120 min during the OGTT. Seventy-eight metabolic measures were analysed at each time point for a discovery cohort of 4745 middle-aged Finnish individuals and a replication cohort of 595 senior Finnish participants. We assessed the metabolic changes in response to glucose ingestion (percentage change in relative to fasting baseline) across the four time points and further compared the response profile between five groups with different levels of IR and glucose intolerance. Further, the differences were tested for covariate adjustment, including gender, body mass index, systolic blood pressure, fasting, and 2-h glucose levels. The groups were defined as insulin sensitive with normal glucose (IS-NGT), insulin resistant with normal glucose (IR-NGT), impaired fasting glucose (IFG), impaired glucose tolerance (IGT), and new diabetes (NDM). IS-NGT and IR-NGT were defined as the first and fourth quartile of fasting insulin in NGT individuals.

**Results:**

Glucose ingestion induced multiple metabolic responses, including increased glycolysis intermediates and decreased branched-chain amino acids, ketone bodies, glycerol, and triglycerides. The IR-NGT subgroup showed smaller responses for these measures (mean + 23%, interquartile 9–34% at 120 min) compared to IS-NGT (34%, 23–44%, *P* < 0.0006 for difference, corrected for multiple testing). Notably, the three groups with glucose abnormality (IFG, IGT, and NDM) showed similar metabolic dysregulations as those of IR-NGT. The difference between the IS-NGT and the other subgroups was largely explained by fasting insulin, but not fasting or 2 h glucose. The findings were consistent after covariate adjustment and between the discovery and replication cohort.

**Conclusions:**

Insulin-resistant non-diabetic individuals are exposed to a similar adverse postprandial metabolic milieu, and analogous cardiometabolic risk, as those with type 2 diabetes. The wide range of metabolic abnormalities associated with IR highlights the necessity of diabetes diagnostics and clinical care beyond glucose management.

## Background

Diabetes affects approximately 1 in 11 adults worldwide, and people with diabetes are at a twofold excess risk for cardiovascular disease (CVD) [1, 2]. A decline in insulin sensitivity is an early sign of susceptibility to type 2 diabetes, typically manifested as elevated levels of fasting insulin [3]. Insulin is a key regulator of glucose metabolism by promoting glucose uptake in peripheral tissues and inhibiting glucose production in the liver [4]. Insufficient insulin action results in increased fasting glucose and eventually leads to overt type 2 diabetes [4]. Insulin resistance (IR) is also linked to the development of cardiometabolic complications, the risk arising already prior to the onset of type 2 diabetes [5, 6]. Studies in the fasting state have identified a cluster of biomarkers robustly associated with IR and predisposing to increased risk for CVD [3, 5, 6]. In the modern society, however, people spend most of their waking hours at a postprandial state, yet we are not aware of epidemiological studies on non-fasting metabolism in representative cohorts.

An oral glucose tolerance test (OGTT) assesses an individual’s ability to clear circulating glucose after an ingestion of a 75-g glucose bolus taken after an overnight fast. An OGTT induces a transition from fasting to feeding, and subsequent changes in various metabolic nutrients occur as the body makes adjustments to achieve glucose homeostasis [7]. It is thus feasible to expect that individuals with impaired insulin action are likely to display a widespread systemic abnormality beyond glucose. Although the dynamics of insulin and glucose during an OGTT in both healthy and insulin-resistant individuals are well studied [8, 9], much less is known on other, particularly emerging cardiometabolic biomarkers, for example, lipoprotein lipid profiles, amino acids, ketone bodies, and inflammatory markers [10, 11].

Metabolic profiling, simultaneously measuring multiple metabolic measures, has been frequently used in studying metabolic dysregulations in the fasting state. Previous studies have revealed that higher fasting ketone bodies, branched-chain amino acids, and aromatic amino acids are predictive for future type 2 diabetes [10, 12]. Similarly, higher concentration of very-low-density lipoprotein (VLDL) particles and increased triglycerides are associated with higher risk of cardiovascular diseases [13]. In particular, recent genetic studies have suggested that disturbed branched-chain amino acid metabolism and increased triglycerides are on the causal path of cardiometabolic diseases [14, 15]. Metabolic profiling has also been applied to assess the metabolic changes during OGTT in small studies. For example, amino acids, ketone bodies, and triglycerides are decreased during an OGTT and some of these changes seem to be blunted in obese and insulin-resistant individuals [7, 16–21]. However, all these studies have been limited in their sample size (up to a few hundred individuals) and often spanned only two time points (pre- and post-OGTT).

In this study, we performed an OGTT across 4 time points and quantified 78 metabolic measures for a total of 5340 individuals (over 21,000 serum samples) from 2 independent population-based cohorts. Our aims were (1) to comprehensively characterise systemic metabolic responses to oral glucose in large scale and (2) to investigate how insulin resistance is associated with postprandial metabolic dysregulation across multiple clinical categories of glucose intolerance. To our knowledge, this is the first population-based large-scale metabolomics time-series study of an OGTT, providing new insights into the metabolic consequences of insulin resistance in non-fasting conditions.

## Methods

### Study population

The Northern Finland Birth Cohort 1966 (NFBC66) was initiated to study factors affecting preterm birth and subsequent morbidity in the two northernmost provinces in Finland [22]. It included 12,058 children born alive, comprising 96% of all births during 1966 in the region. The participants were further followed up at the age of 1, 14, 31, and 46 years. Data collection conducted in 2012 at their age of 46, including clinical examination and serum sampling, was available for 5839 individuals. Among them, 4745 study participants, who were free of prior diagnosed diabetes, underwent metabolic profiling of OGTT serum samples (97% had 4 time points), and had information on baseline fasting insulin and glucose, were used in this study.

The Oulu1945 cohort studies ageing populations in Oulu, Finland. It was started in 2000 and was originally comprised of 1400 individuals born in 1945. In the follow-up study conducted in 2015, data collection including clinical examination and serum sampling was available for 717 participants. Among them, 595 participants who were free of prior diagnosed diabetes, underwent metabolic profiling of OGTT samples (92% had 4 time points), and had data on baseline fasting insulin and glucose were included.

### Clinical assessment

Subjects underwent a 2-h, 75-g OGTT after an overnight fasting. Blood samples were obtained at 0 (fasting baseline, right before glucose ingestion), 30, 60, and 120 min during the OGTT. Plasma glucose were analysed by an enzymatic dehydrogenase method (Advia 1800, Siemens Healthcare Diagnostics, Tarrytown, NY, USA) and serum insulin by a chemiluminometric immunoassay (Advia Centaur XP, Siemens Healthcare Diagnostics, Tarrytown, NY, USA). Insulin resistance was estimated by fasting insulin, homeostasis model assessment of insulin resistance (HOMA-IR), and insulin sensitivity index-Matsuda (ISI-Matsuda). First-phase insulin secretion, an index of beta-cell function, was measured by insulinogenic index. The formulas for these models are shown in the legend for Table 1.

**Table 1:** Characteristics of the Northern Finland Birth Cohort 1966

	Without glucose abnormality			With glucose abnormality		
	All NGT	IS-NGT	IR-NGT	IFG	IGT	NDM
*N*	2847	708	713	1380	412	106
Age [year]	46.7 [46.2–47.1]	46.5 [46.2–46.9]	46.8 [46.4–47.3]	46.6 [46.2–47.1]	46.7 [46.3–47.2]	46.8 [46.3–47.2]
Men [%]	34	33	38	61	48	63
BMI [kg/m^2^]	24.9 [22.7–27.6]	23.1 [21.6–25.1]	27.8 [25.1–30.9]	27.0 [24.6–29.9]	29.2 [26.3–32.4]	30.0 [27.3–33.3]
Systolic blood pressure [mmHg]	120 [111–131]	118 [109–128]	125 [114–135]	128 [118–138]	130 [122–142]	136 [124–150]
Diastolic blood pressure [mmHg]	82 [75–89]	79 [73–86]	85 [78–93]	86 [80–93]	90 [82–97]	94 [86–100]
Triglycerides [mmol/L]	1.0 [0.7–1.3]	0.8 [0.6–1.1]	1.3 [1.0–1.7]	1.3 [0.9–1.8]	1.6 [1.1–2.2]	1.7 [1.3–2.2]
LDL cholesterol [mmol/L]	1.8 [1.5–2.2]	1.7 [1.5–2.2]	1.9 [1.6–2.4]	2.0 [1.6–2.4]	1.9 [1.6–2.3]	2.0 [1.7–2.3]
HDL cholesterol [mmol/L]	1.7 [1.4–1.9]	1.8 [1.6–2.0]	1.5 [1.3–1.7]	1.5 [1.3–1.8]	1.4 [1.2–1.7]	1.4 [1.1–1.6]
Fasting insulin [mU/L]	6.5 [4.6–9.5]	3.6 [3.0–4.1]	12.2 [10.6–14.9]	9.8 [6.9–13.5]	12.4 [8.3–18.3]	15.5 [10.5–24.4]
2 h insulin [mU/L]	37.0 [25.2–53.2]	25.2 [17.9–34.2]	59.9 [41.9–92.8]	47.9 [31.6–77.6]	120.4 [68.8–187.4]	129.9 [66.0–195.5]
Fasting glucose [mmol/L]	5.2 [5.0–5.4]	5.0 [4.8–5.3]	5.3 [5.1–5.4]	5.8 [5.7–6.0]	5.7 [5.4–6.1]	6.9 [6.3–7.2]
2 h glucose [mmol/L]	5.2 [4.6–5.9]	4.9 [4.3–5.6]	5.6 [4.9–6.3]	5.9 [5.1–6.6]	8.6 [8.1–9.2]	11.5 [8.4–12.6]
HOMA-IR	1.5 [1.0–2.2]	0.8 [0.7–0.9]	2.8 [2.5–3.5]	2.5 [1.8–3.5]	3.2 [2.0–4.7]	4.5 [3.2–7.6]
ISI (Matsuda)	6.3 [4.4–8.6]	10.6 [8.9–12.7]	3.4 [2.6–4.2]	3.8 [2.6–5.5]	2.5 [1.7–3.9]	1.9 [1.2–3.0]
Insulinogenic index	21.7 [13.3–38.2]	15.7 [9.9–26.2]	32.2 [20.2–49.2]	20.7 [12.6–33.2]	18.0 [10.5–30.2]	10.8 [7.6–20.2]

Values are median [interquartile range]. Characteristics for the replication cohort Oulu45 is shown in Additional file 1: Table S1

*Abbreviations*: *BMI* body mass index, *NGT* normal glucose tolerance, *IFG* isolated impaired fasting glucose, *IGT* isolated impaired glucose tolerance, *NDM* new type 2 diabetes, *IS-NGT* insulin-sensitive individuals within NGT (at the first quartile of fasting insulin within NGT), *IR-NGT* insulin-resistant individuals within NGT (at the top quartile of fasting insulin within NGT)

HOMA-IR = fasting glucose (mmol/L) × fasting insulin (mIU/L)/22.5

ISI-Matsuda = 10,000/square root of [fasting glucose (mg/dL) × fasting insulin (mIU/L) × mean glucose × mean insulin during OGTT]

Insulinogenic index = (Insulin_30 (mIU/L)_ − Insulin_0_)/(Glucose_30(mmol/L)_ − Glucose_0_) during OGTT

**Fig. 1:**
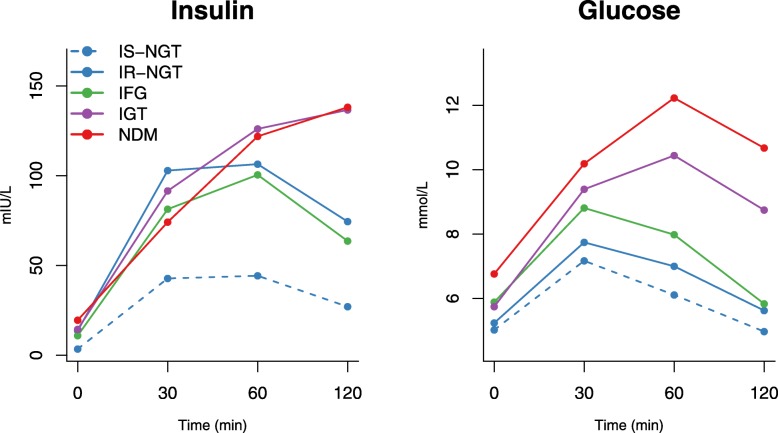
Mean concentration of insulin and glucose at 0, 30, 60, and 120 min during an oral glucose tolerance test. Insulin and glucose trajectories for insulin-sensitive subgroup of normal glucose tolerance (IS-NGT, dashed blue, *n* = 708), insulin-resistant subgroup of normal glucose tolerance (IR-NGT, solid blue, *n* = 713), impaired fasting glucose (IFG, green, *n* = 1380), impaired glucose tolerance (purple, *n* = 412), and newly-diagnosed type 2 diabetes (red, NDM, *n* = 106) are shown. IS-NGT was defined as the bottom quartile of fasting insulin within NGT, and IR-NGT was defined as the top quartile. The dots denote mean absolute concentrations. Interquartile ranges are listed in Table 1

According to an individual’s insulin resistance status and the American Diabetes association 2003 criteria [8], participants were classified into five groups (Table 1, Fig. 1, and Additional file 1: Table S1):

1. Insulin-sensitive subgroup of normal glucose tolerance (IS-NGT, fasting insulin at the bottom quartile of NGT and fasting glucose < 5.6 mmol/L and 2-h glucose < 7.8 mmol/L)
2. Insulin-resistant subgroup of normal glucose tolerance (IR-NGT, fasting insulin at the top quartile of NGT and fasting glucose < 5.6 mmol/L and 2-h glucose < 7.8 mmol/L)
3. Impaired fasting glucose (IFG, fasting glucose between 5.6 and 6.9 mmol/L and 2-h glucose < 7.8 mmol/L)
4. Impaired glucose tolerance (IGT, fasting glucose ≤ 6.9 mmol/L and 2-h glucose between 7.8 and 11.0 mmol/L)
5. New onset of type 2 diabetes (NDM, fasting glucose ≥ 7.0 mmol/L or 2-h glucose ≥ 11.1 mmol/L)

### Metabolic profiling

The human serum metabolome is dominated by hydrophobic lipid-like molecules, including diglycerides, triglycerides, phospholipids, fatty acids, steroids, and steroid derivatives [23]. These lipids are packed in various lipoprotein particles, e.g. VLDL, intermediate-density lipoprotein (IDL), low-density lipoprotein (LDL), and high-density lipoprotein (HDL). Other metabolites found in high abundance in serum include amino acids, glucose, lactate, and several waste or catabolic by-products, such as urea and creatinine [23]. Here, a nuclear magnetic resonance (NMR) spectroscopy metabolomics platform was used to measure all the detectable lipids and metabolites in a non-selective way. The high-throughput NMR metabolomics platform was applied to quantify over 200 lipid and metabolite measures from serum samples collected at 0, 30, 60, and 120 min during an OGTT challenge. The platform applies a single experimental setup, which allows for simultaneous quantification of standard clinical lipids, 14 lipoprotein subclasses, and individual lipids (triglycerides, phospholipids, free and esterified cholesterol) transported by these particles, multiple fatty acids, glucose and various glycolysis precursors, ketone bodies, and amino acids in absolute concentration units [24–26]. As the total lipids and individual lipids within the same lipoprotein subclass are highly correlated [27], we chose a priori to analyse the total lipids in the 14 subclasses and limit specific lipids for the 4 major fractions (VLDL, IDL, LDL, and HDL). These together with all the fatty acids and non-lipid measures provided by this platform, in total 77 measures, were used in the present study. A similar metabolic panel has been widely applied in previous studies [3, 28, 29].

### Statistical analyses

In total, 78 metabolic measures were used in the analyses. Of those, 77 were measured by NMR metabolomics and glucose by a clinical assay. Insulin was treated as an exposure in this study. All analyses were undertaken in the R programming environment (version 3.5.1). Primary analyses were conducted using NFBC66, and key results were replicated in Oulu1945.

To study the physiological response to an OGTT, metabolic trajectories for NGT individuals were reported. Metabolic trajectories were calculated as percentage changes in relative to baseline concentration at 30, 60, and 120 min, respectively, e.g. (Concentration_120m_ − Concentration_0m_)/Concentration_0m_ × 100%. In the formula, metabolic concentrations are in their original units, e.g. mmol/L. The significance of a change was evaluated via paired *t* test by comparing the metabolite concentration at post-load time points against the fasting baseline. The analyses were repeated for men and women separately. Due to the correlated nature of the metabolic measures, 19 principle components were able to explain 95% variation of the 78 measures; therefore, *P* < 0.05/19/4 = 0.0006 was considered statistically significant after correcting for multiple comparisons (corrected for 19 independent components and across 4 time points) [30, 31].

To assess whether metabolic trajectories would be different across the groups, two-way ANOVA was used, with metabolite change (%) as the response, time points × groups as the interaction term, and gender as the covariate. In total, 60 out of 78 measures showed significant interaction of time points and groups, suggesting the metabolic trajectories would be different between the groups for these measures (Additional file 2: Table S2). *t* tests were further used to compare the metabolic trajectories between IR-NGT and IS-NGT across the 78 measures. For those metabolic measures that showed significant differences between IR-NGT and IS-NGT, we further assessed their differences between IR-NGT and those with IGT or NDM.

In addition, sensitivity analyses were conducted to assess the effect of potential covariates for those measures that showed significant differences between IR-NGT and IS-NGT. Linear regression models were used to quantify the metabolic differences between the groups, using 2-h change in metabolite concentration as the response variable and group category as the independent variable. Four sets of covariates were used: (1) sex, (2) sex + BMI + systolic blood pressure, (3) sex + baseline glucose + 2-h glucose, and (4) sex + fasting insulin. Metabolite concentrations at baseline and 2 h were log-transformed, and the changes between the baseline and 2 h were scaled to baseline SD.

## Results

Two population cohorts were used to study the metabolic changes during an OGTT. The primary analyses were conducted in 4745 individuals in the NFBC66 (mean age 47 years, 44% men, Table 1), and the key results were replicated in 595 participants in the Oulu1945 (mean age 69 years, 41% men, Additional file 1: Table S1). Among the participants in the NFBC66, 60% of individuals had normal fasting and 2-h glucose (NGT), 29% had impaired fasting glucose (IFG), and 11% had impaired 2-h glucose tolerance (IGT or NDM). Although NGT individuals are generally considered healthy, the IR-NGT subgroup had over 3 times higher fasting insulin than the IS-NGT. After glucose ingestion, these insulin-resistant individuals secreted even more insulin in the early phase (30 min), yet they were still unable to restore glucose levels back to the pre-OGTT levels after 2 h (Table 1 and Fig. 1). The IR-NGT individuals were also more likely to be male and had higher BMI, blood pressure, and fasting triglycerides and lower HDL cholesterol (Table 1). Similar characteristics were observed for IFG, IGT, and NDM, and their fasting insulin levels were comparable to IR-NGT, ranging from 2.7 to 4.3 times more than IS-NGT.

### Metabolic trajectories under normal glucose tolerance

**Fig. 2:**
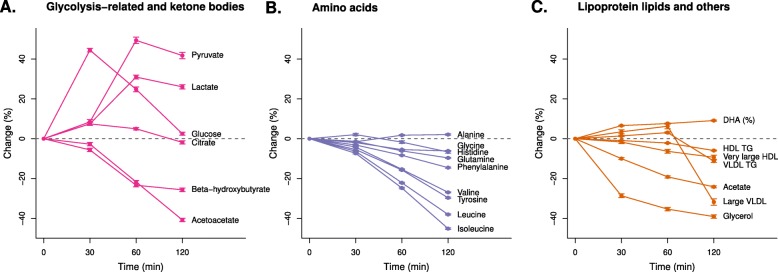
Selected metabolic changes in response to an oral glucose tolerance test in individuals with normal glucose tolerance. The dots and error bars denote mean percent change and 95%CI. Percent change is defined as the absolute change in relative to baseline. **a** Glycolysis-related and ketone bodies. **b** Amino acids. **c** Lipoprotein lipids and others

Selected responses to an OGTT for the NGT individuals are summarised in Fig. 2 (*P* < 0.0006 at any time point), and results for all measures are available in Additional file 1: Figure S1 and Additional file 2: Table S3. During the OGTT, glycolysis-related metabolic measures (pyruvate and lactate) were primarily increased during 30 and 60 min (peaking at 60 min with 49% [interquartile 19%, 74%] and 31% [14%, 47%], respectively), lagging approximately 30 min behind the glucose rise (Fig. 2a). A smaller increase was seen with citrate (peaking with 7% [− 2%, 15%] at 30 min). On the other hand, ketone bodies beta-hydroxybutyrate and acetoacetate were continuously reduced after glucose ingestion and lowered by 26% (8%, 42%) and 41% (29%, 56%) at 120 min, respectively. Similarly, almost all amino acids were decreased during the OGTT, except for alanine (Fig. 2b). Branched-chain (isoleucine, leucine, and valine) and aromatic amino acids (phenylalanine and tyrosine) were decreased (15 to 45%) more than the other amino acids (6 to 10%) at 120 min. Acetate and glycerol were decreased throughout the OGTT and reduced by 24% (16%, 33%) and 39% (25%, 55%) at 120 min, respectively (Fig. 2c). Changes in lipids and fatty acids were generally smaller in comparison to the aforementioned non-lipid measures (Fig. 2 and Additional file 1: Figure S1A). The largest changes in lipids were seen for the total lipids in extremely large, very large, large, and medium VLDL particles, with 11 to 32% reduction at 120 min, after initial increases at 60 min (e.g. large VLDL in Fig. 2c). All HDL subclass measures were suppressed during the OGTT, with a 2 to 9% decrease at 120 min (e.g. very large HDL in Fig. 2c). Interestingly, circulating triglycerides in all main lipoprotein particles, VLDL, IDL, LDL, and HDL, were decreased at 120 min (1 to 11%, e.g. VLDL-TG and HDL-TG in Fig. 2c). Inconsistent and small changes were seen in the corresponding cholesterol concentrations (see Additional file 1: Figure S1A for details).

### Metabolic trajectories under insulin resistance

**Fig. 3:**
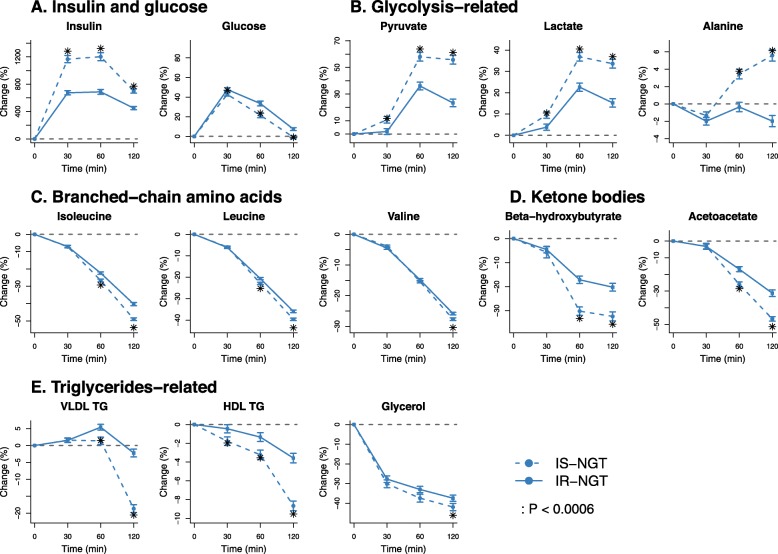
Metabolic trajectoires compared between insulin-resistant and insulin-sensitive individuals in the normal glucose tolerance group. IS-NGT, indiviudals with normal glucose tolerance and in the first quartile of fasting insulin (*n* = 708); IR-NGT, individuals with normal glucose tolerance and in the top quartile of fasting insulin (*n* = 713). The dots and error bars denote mean percentage changes and 95% confidence intervals, respectively. The asterisk denotes that there are signficiant differences between IS-NGT and IR-NGT at corresponding time point. **a** Insulin and glucose. **b** Glycolysis-related. **c** Branched-chain amino acids. **d** Ketone bodies. **e** Triglycerides-related

Metabolic trajectories of IR-NGT were compared to those of IS-NGT (Fig. 3). The analyses were restricted to individuals with normal glucose tolerance to rule out any secondary effects from hyperglycaemia. Full results for all 78 measures are available in Additional file 1: Figure S2 and Additional file 2: Table S4. Pronounced differences were observed in multiple metabolic pathways including glycolysis-related metabolites, branched-chain amino acids, ketone bodies, and triglyceride-related measures (Fig. 3b–e). Typical differences were initially small at 30 min and became more pronounced from 60 min onwards (except for insulin and glucose). At 120 min, the IR-NGT individuals showed higher increase in glucose yet smaller increase in pyruvate, lactate, and alanine levels. Also, they displayed smaller decrease in branched-chain amino acids and ketone bodies as well as triglyceride-related measures. Overall, the changes at 120 min across these measures (Fig. 3b–e) were 34% (interquartile 23–44%) in IS-NGT, whereas only 23% (9–34%) in IR-NGT. These differences were statistically significant with *P* < 0.0006 (Additional file 1: Figure S2B). The results were consistent when stratified by sex (Additional file 1: Figure S3). Also, the results were similar when we compared the top and bottom quartiles of the HOMA-IR and 1/Matsuda indices (Additional file 1: Figure S4).

### Metabolic trajectories under prediabetes and diabetes

**Fig. 4:**
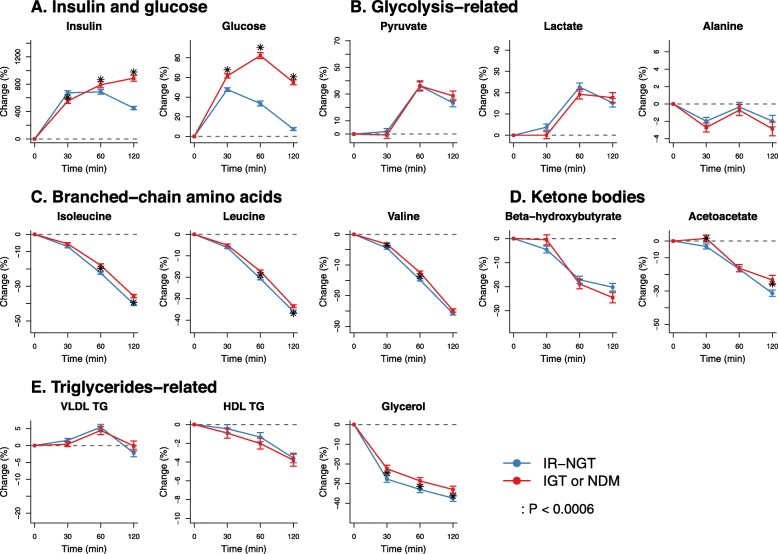
Metabolic trajectories compared between insulin-resistant individuals in the normal glucose tolerance group (blue) and those with 2-h impaired glucose tolerance (red). IR-NGT, indiviudals with normal glucose tolerance and in the top quartile of fasting insulin (*n* = 713); IGT/NDM, Individuals with 2-h impaired glucose tolerance, including those with impaired glucsoe tolerance and new onset of type 2 diabetes (*n* = 518). The dots and error bars denote mean percentage changes and 95% confidence intervals, respectively. The asterisk denotes that there are signficiant differences between IR-NGT and those with IGT or NDM at corresponding time point. **a** Insulin and glucose. **b** Glycolysis-related. **c** Branched-chain amino acids. **d** Ketone bodies. **e** Triglycerides-related

Figure 4 (Additional file 2: Table S5) presents the comparison of the metabolic trajectories in individuals with 2-h impaired glucose tolerance (IGT or NDM) and those of IR-NGT. Although large differences in glucose responses were observed by definition, these two groups showed marginal differences in metabolic responses in glycolysis products, branched-chain amino acids, ketone bodies, and triglyceride-related measures (Fig. 4b–e). In addition, the IFG individuals who had normal 2-h glucose response but high fasting glucose (5.9 vs 5.2 mmol/L in IFG and IR-NGT) also showed marginal differences in metabolic trajectories compared to those of IR-NGT (Additional file 1: Figure S5). The metabolic trajectories in percent change and absolute concentrations across all five individual groups (IS-NGT, IR-NGT, IFG, IGT, and NDM) are shown in Additional file 1: Figures S6 and S7. Results corresponding to those shown in Figs. 2, 3, and 4 for the discovery cohort NFBC66 are replicated in the Oulu1945 cohort (see Additional file 1: Figure S8 for detailed results).

### Metabolic responses associated with IR with or without glucose abnormality

**Fig. 5:**
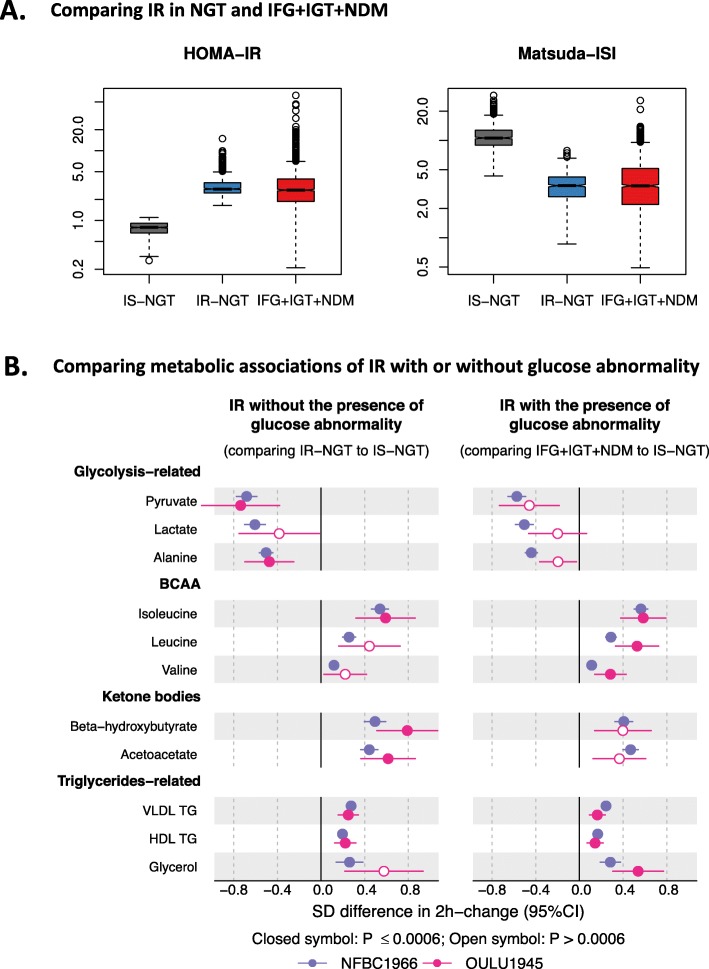
Summary and replication. **a** Estimated insulin resistance in IS-NGT (grey), IR-NGT (blue), and pooled of IFG, IGT, and NDM (red) in NFBC66. **b** Two-hour metabolic responses associated with IR with or without glucose abnormality in NFBC66 (purple) and replicated in Oulu45 (red). Groups were compared by linear regression models with the 2-h concentration change as the response variable. Baseline and 2-h metabolite concentrations were log-transformed, and the changes between 2-h and baseline metabolite concentrations were scaled to baseline SD. Group sizes within NFBC66: *n* = 708 in IS-NGT, *n* = 713 in IR-NGT, and *n* = 1898 in combined IFG, IGT, and NDM. Group sizes within Oulu1945: *n* = 62 in IS-NGT, *n* = 64 in IR-NGT, and *n* = 343 in combined IFG, IGT, and NDM

**Fig. 6:**
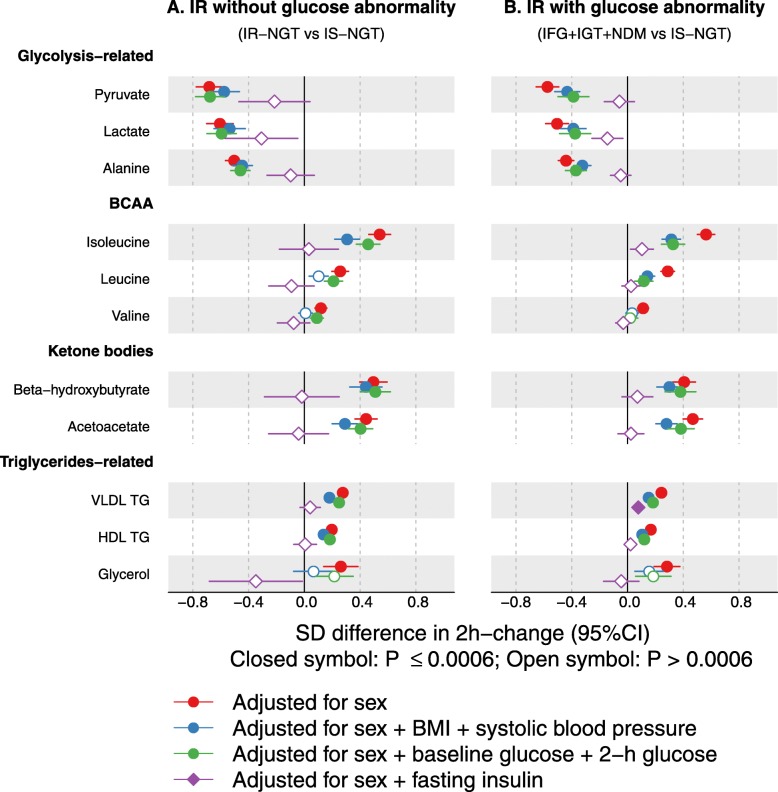
Group comparison adjusted for baseline factors in the NFBC66 cohort. **a** Differences in 2-h changes between the IR-NGT (*n* = 713) and the IS-NGT group (*n* = 708). **b** Differences in 2-h changes in the combined IFG, IGT, and NDM (*n* = 1898) and the IS-NGT group (*n* = 708). Groups were compared by linear regression models with the 2-h concentration change as the response variable. Baseline and 2-h metabolite concentrations were log-transformed, and the changes between 2-h and baseline metabolite concentrations were scaled to baseline SD. Insulin was log-transformed

Figure 5a displays the distributions of insulin resistance measured by HOMA-IR and Matsuda index in individuals with IS-NGT, IR-NGT, and IFG + IGT + NDM. Despite the IFG + IGT + NDM group having impaired glucose metabolism by definition, these individuals together with the IR-NGT group showed comparable HOMA-IR and Matsuda indices. Interestingly, these two groups also showed similar differences in the 2-h metabolite responses when compared to the IS-NGT group (Fig. 5b). This was consistently observed in the two independent cohorts. The metabolic differences associated with IR-NGT and IFG + IGT + IGT remained the same or became slightly attenuated after adjusting for BMI, systolic blood pressure, baseline glucose, and 2-h glucose (Fig. 6). By contrast, the associations were substantially attenuated to almost null after adjusting for fasting insulin. Similar results were observed when IFG, IGT, and NDM were individually compared to IS-NGT with the adjustments (Additional file 1: Figure S9).

Lastly, we observed distinctive patterns in fasting metabolic concentrations and the 2-h metabolite responses (Additional file 1: Figures S7 and S10). Branched-chain amino acids and triglycerides in IR individuals were higher at baseline and exhibited less decrease at 2 h, compared to the IS-NGT group. Glycolysis-related measures were higher in IR individuals at baseline, but increased less at 2 h, whereas ketone bodies seemed to be lower at baseline, but decreased less at 2 h compared to the IS-NGT group.

## Discussion

We profiled four time points of OGTT data for in total 5340 Finnish individuals from 2 independent cohorts to obtain new large-scale population-based information on how insulin resistance is associated with a systemic post-load metabolic dysregulation. These changes include adverse modifications in multiple cardiometabolic biomarkers suggesting that insulin resistance may underlie the shared susceptibility to diabetes and CVD also in the post-load milieu. Our study is important because most people spend a significant amount of their daily lives in a postprandial state—this aspect of insulin resistance has not been captured in previous metabolomics studies of fasting samples. The results also carry practical significance: we found that IR-associated metabolic aberrations exist already in participants with normal glucose tolerance (with implications for CVD risk) and are similar in extent to those observed in type 2 diabetes.

The large sample size and multiple metabolomics time points allowed us to obtain accurate and systemic understanding of the expected metabolic changes in response to glucose ingestion in people with normal glucose tolerance. Our temporal data on the 2-h changes were consistent with previous small studies with pre- and post-OGTT measures and support the known action of insulin in promoting glycolysis metabolism (pyruvate and lactate) and suppression of ketogenesis (ketone bodies), proteolysis (amino acids), and lipolysis (glycerol) [4, 7, 18, 20]. Additionally, our results showed that glucose ingestion also reduces the circulating concentration of triglycerides in VLDL particles after the initial increase during the first 60 min. This may reflect a complex balance of hepatic triglyceride production between increased conversion from excess glucose and reduced re-esterification from free fatty acids (as a result of reduced lipolysis) [4]. A general observation is that different metabolic pathways were differentially affected. For example, concentrations of glycolysis-related measures peaked within 2 h, whilst most other measures (e.g. amino acids, ketone bodies, and triglycerides) continuously decreased during the 2 h and had an evident trend afterwards.

The extensive metabolic data demonstrate that insulin-resistant individuals had systematically smaller relative metabolic responses in comparison to the insulin-sensitive ones. Some of these blunted changes have been previously reported for insulin-resistant or obese individuals separately in small studies, e.g. for lactate [7, 20], beta-hydroxybutyrate [7, 20], isoleucine [7, 20], glycerol [7], and VLDL-TG [16, 18]. Interestingly, the metabolic measures which showed blunted changes in insulin-resistant individuals in this study have been also associated with insulin resistance in the fasting state [28]. It has been suggested that insulin resistance is associated with higher fasting glycolysis-related measures and greater fasting concentrations of branched-chain amino acids, glycerol, and triglycerides [28]. Prospective studies have suggested that the associated metabolic dysregulations at fasting state are predictive of future cardiometabolic risk [10, 11, 29, 32]. Further, recent Mendelian randomisation analyses have indicated a causal link from insulin resistance to higher branched-chain amino acids and triglycerides in the fasting state [3]. Our results here underline the possibility that fasting concentrations may also reflect the insufficient suppression of branched-chain amino acids and triglycerides in the postprandial state in the insulin-resistant individuals. Regardless of the exact sequence of events, this study provides new evidence that insulin-resistant individuals are at greater cardiometabolic risk both in the fasting and post-load settings.

The comparison between IR-NGT and IS-NGT addressed the differences in IR whilst having normal glucose metabolism. We also performed a mirror experiment where we compared the metabolic trajectories of IFG, IGT, and NDM to IR-NGT (varying glucose levels but minimising the differences in IR). Interestingly, we found similar metabolic dysregulations in individuals with prediabetes and diabetes to those of insulin-resistant individuals with normal glucose metabolism. These findings suggest limited impact of glucose on these metabolic associations. This interpretation is reinforced by our adjusted analyses: the metabolic dysregulations appear to be exclusively driven by insulin resistance but not fasting or 2-h glucose. Type 2 diabetes, characterised by increased circulating glucose concentrations, is a known risk factor for CVD. However, a meta-analysis of prospective studies found only a marginal association between circulating glucose and CVD outcomes [2]. Consistently, a meta-analysis of over 300 trials found limited evidence to support glucose-lowering drugs would reduce the risk of cardiovascular disease and all-cause mortality in patients of established type 2 diabetes [33]. By contrast, individuals at the stage of IR-NGT or prediabetes are reported to have higher risk of CVD [6, 34]. Taking these together, it seems that long-term exposure for the metabolic consequences of insulin resistance across multiple tissues would account for the concerting development of type 2 diabetes and cardiometabolic complications [5, 6]. Our study revealed that glucose-independent postprandial dysfunction might be a novel component of this exposure that is hitherto poorly recognised as a potential interventional target.

Large-scale population studies and multiple time points of metabolomics data gave us a unique opportunity to study the systemic metabolic trajectories across multiple clinical glucose categories. Analyses with multiple testing, multivariate adjustments, and replication in an independent cohort all point towards the robustness of the current findings. The associations of insulin resistance with the metabolic changes were consistent when assessed across three different surrogate markers of insulin resistance. However, we acknowledge that insulin resistance markers may reflect a composite state of insulin sensitivity levels of multiple tissues. In order to understand the metabolic signatures of specific tissues, further experiments are required. In addition, the results were coherent whether the metabolic changes were assessed via relative or absolute concentration changes. The associations remained similar between men and women, between middle-aged and older individuals, and also between those with or without the presence of glucose abnormality. However, ethnic and socioeconomic context should be taken into account when extending these results to other populations. The OGTT corresponds to the ingestion of sugary drinks, but not mixed meals, and thus, these results should not be generalised to post-meal metabolic responses.

## Conclusions

In conclusion, our results highlight the detrimental effects of insulin resistance on systemic metabolism after glucose ingestion. The population health impact of these metabolic consequences is likely substantial given the spurious and energy-dense eating patterns in the modern world, i.e. people are mostly living in a non-fasting state and consume high amounts of added sugar and refined carbohydrates. The observed metabolic effects manifest very early on, and these findings suggest new avenues to understand the increased CVD risk in insulin resistance and diabetes. It might therefore be beneficial if diabetes diagnostics and clinical care would be extended beyond glucose management. We call for better recognition of postprandial dysfunction beyond glucose tolerance categories as an important cardiometabolic risk factor, and new preventive efforts and strategies to reverse all aspects of metabolic dysregulation. We maintain that this is particularly important at the early stages of insulin resistance, and may also hold untapped therapeutic opportunities.

## Supplementary information

**Additional file 1: Table S1.** Characteristics of the Oulu1945 cohort. **Figure S1.** A. Changes in 78 metabolic measures in response to an oral glucose tolerance test. B. Corresponding significance levels for A. **Figure S2.** A. Difference in metabolic changes between IR-NGT and IS-NGT. B. Corresponding significance level for A. **Figure S3.** Differences in metabolic changes associated with insulin resistance stratified by sex. **Figure S4.** Differences in metabolic changes associated with fasting insulin, HOMA-IR and Matsuda-index. **Figure S5.** Metabolic trajectories compared between insulin resistant individuals of normal glucose tolerance group to those with impaired fasting glucose. **Figure S6.** Metabolic trajectories for individuals with IS-NGT, IR-NGT, IFG, IGT, and NDM. **Figure S7.** Absolute metabolic concentrations at 0 ,30 ,60 and 120 minute for IS-NGT, IRNGT, IFG, IGT and NDM. **Figure S8.** Replication in Oulu1945. **Figure S9.** Group comparisons adjusted with different factors. **Figure S10.** Difference in baseline metabolite concentration (left) and 2-h metabolic change (right) comparing the groups to the reference group (IS-NGT).

**Additional file 2: Table S2.** Interaction test of time groups and glucose groups for 78 metabolic measrues, adjusted for sex. **Table S3.** Change (%) in relative to baseline at 30, 60, 120 minute for five individual groups across 78 metabolic measures. **Table S4.** Differences in metabolite changes (%) between IR-NGT and IS-NGT. **Table S5.** Differences in metabolite changes (%) between IR-NGT and those with IGT or NDM.

## Data Availability

Data are available for researchers who meet the criteria for access to confidential data according to the rules of each individual cohort and can be requested from the Institutional Data Access Committees of the Northern Finland Birth Cohort Study and the Oulu1945 study (University of Oulu, Finland).

## Abbreviations

BMI: Body mass index
CVD: Cardiovascular disease
HDL: High-density lipoprotein
HOMA-IR: Homeostasis model assessment of insulin resistance
IDL: Intermediate-density lipoprotein
IFG: Impaired fasting glucose
IGT: Impaired glucose tolerance
IR: Insulin resistance
IR-NGT: Insulin resistance subgroup of normal glucose tolerance
ISI-Matsuda: Insulin sensitivity index-Matsuda
IS-NGT: Insulin-sensitive subgroup of normal glucose tolerance
LDL: Low-density lipoprotein
NDM: New onset of type 2 diabetes
NFBC66: The Northern Finland Birth Cohort 1966
NMR: Nuclear magnetic resonance
OGTT: Oral glucose tolerance test
VLDL: Very-low-density lipoproteins
