# Supplementary material for: Insulin resistance and systemic metabolic changes in oral glucose tolerance test in 5340 individuals: an interventional study
Source: BMC Med. 2019 Nov 29;17:217. doi: 10.1186/s12916-019-1440-4 (PMC6883544; doi:10.1186/s12916-019-1440-4)
Supplement: Supplementary file 1 — Additional file 1: Table S1. Characteristics of the Oulu1945 cohort. Figure S1. A. Changes in 78 metabolic measures in response to an oral glucose tolerance test. B. Corresponding significance levels for A. Figure S2. A. Difference in metabolic changes between IR-NGT and IS-NGT. B. Corresponding significance level for A. Figure S3. Differences in metabolic changes associated with insulin resistance stratified by sex. Figure S4. Differences in metabolic changes associated with fasting insulin, HOMA-IR and Matsuda-index. Figure S5. Metabolic trajectories compared between insulin resistant individuals of normal glucose tolerance group to those with impaired fasting glucose. Figure S6. Metabolic trajectories for individuals with IS-NGT, IR-NGT, IFG, IGT, and NDM. Figure S7. Absolute metabolic concentrations at 0 ,30 ,60 and 120 minute for IS-NGT, IRNGT, IFG, IGT and NDM. Figure S8. Replication in Oulu1945. Figure S9. Group comparisons adjusted with different factors. Figure S10. Difference in baseline metabolite concentration (left) and 2-h metabolic change (right) comparing the groups to the reference group (IS-NGT). [file 12916_2019_1440_MOESM1_ESM.pdf]

Supplementary Materials for:

Insulin resistance and systemic metabolic changes in oral glucose tolerance test in 5,340 individuals: An interventional study

Wang *et al.*

Content

Supplementary Tables

Table S1. Characteristics of the Oulu1945 cohort.

Table S2. Interaction test of time groups and glucose groups for 78 metabolic measures, adjusted for sex.

Table S3. Change (%) in relative to baseline at 30, 60, 120 min for five individual groups across 78 metabolic measures.

Table S4. Differences in metabolite changes (%) between IR-NGT and IS-NGT.

Table S5. Differences in metabolite changes (%) between IR-NGT and those with IGT or NDM.

Tables S2-S5 are in Additional file 2.

Supplementary Figures

Figure S1.

S1A. Changes in 78 metabolic measures in response to an oral glucose tolerance test.

S1B. Corresponding significance levels for Figure S1A.

Figure S2.

S2A. Difference in metabolic changes between IR-NGT and IS-NGT.

S2B. Corresponding significance level for Figure S2A.

Figure S3. Differences in metabolic changes associated with insulin resistance stratified by sex.

Figure S4. Differences in metabolic changes associated with fasting insulin, HOMA-IR and Matsuda-index.

Figure S5. Metabolic trajectories compared between insulin resistant individuals of normal glucose tolerance group to those with impaired fasting glucose.

Figure S6. Metabolic trajectories for individuals with IS-NGT, IR-NGT, IFG, IGT, and NDM.

Figure S7. Absolute metabolic concentrations at 0 ,30 ,60 and 120 minute for IS-NGT, IR-NGT, IFG, IGT and NDM.

Figure S8. Replication in Oulu1945.

Figure S9. Group comparisons adjusted with different factors.

Figure S10. Difference in baseline metabolite concentration (left) and 2-h metabolic change (right) comparing the groups to the reference group (IS-NGT).

Supplementary Tables

Table S1. Characteristics of the Oulu1945 cohort.

		Insulin resistance (fasting insulin)		Prediabetes and Type 2 diabetes		
	NGT	NGT-IS	NGT-IR	IFG	IGT	NDM
N	252	62	64	130	178	35
Age [year]	68.9 [68.5-69.3]	68.9 [68.7-69.3]	68.8 [68.6-69.3]	68.8 [68.4-69.2]	68.8 [68.4-69.3]	68.9 [68.3-69.3]
Men [%]	33	38	30	50	41	74
BMI [kg/m2]	25.5 [23.2-27.9]	23.5 [21.6-25.5]	27.3 [25.4-29.6]	27.0 [24.6-29.9]	28.1 [25.1-30.9]	31.1 [25.8-34.3]
Systolic blood pressure [mmHg]	138 [130-151]	134 [124-152]	143 [128-154]	143 [130-153]	145 [136-161]	150 [141-159]
Diastolic blood pressure [mmHg]	83 [78-90]	82 [77-88]	85 [80-93]	84 [78-91]	88 [82-94]	89 [82-93]
Triglycerides (mmol/L)	1.0 [0.9-1.2]	0.9 [0.8-1.1]	1.2 [0.9-1.4]	1.1 [0.9-1.3]	1.2 [1.0-1.6]	1.3 [1.1-1.7]
LDL cholesterol (mmol/L)	1.6 [1.3-1.9]	1.6 [1.3-1.8]	1.4 [1.2-1.7]	1.5 [1.2-1.9]	1.6 [1.2-1.9]	1.5 [1.0-1.7]
HDL cholesterol (mmol/L)	1.5 [1.3-1.7]	1.7 [1.4-1.8]	1.3 [1.1-1.6]	1.4 [1.2-1.6]	1.4 [1.2-1.6]	1.2 [1.0-1.4]
Fasting insulin [mU/L]	9.8 [7.2-13.1]	5.5 [4.9-6.4]	15.6 [14.4-17.8]	12.2 [9.0-16.9]	14.1 [9.5-19.8]	16.2 [10.2-28.4]
2h insulin [mU/L]	47.2 [31.0-65.5]	31.1 [20.4-42.3]	74.6 [59.0-131.2]	54.9 [40.1-73.9]	122.6 [66.3-175.4]	114.9 [57.8-208.7]
Fasting glucose [mmol/L]	5.2 [5.0-5.4]	5.0 [4.8-5.3]	5.2 [5.0-5.5]	5.9 [5.7-6.1]	5.8 [5.4-6.2]	6.6 [6.2-7.1]
2h glucose [mmol/L]	6.2 [5.6-6.9]	6.0 [5.4-6.6]	6.7 [6.1-7.3]	6.6 [6.0-7.2]	8.8 [8.2- 9.6]	11.3 [9.2-12.6]
HOMA-IR	2.2 [1.6-3.0]	1.2 [1.1-1.4]	3.7 [3.3-4.2]	3.3 [2.4-4.6]	3.7 [2.3-5.1]	4.7 [3.0-8.2]
ISI (Matsuda)	4.2 [3.1-5.7]	7.0 [5.8-8.2]	2.5 [2.0-3.0]	2.9 [2.2-4.1]	2.7 [1.6-3.8]	1.8 [1.2-3.2]
Insulinogenic index	18.3 [11.4-30.5]	11.8 [8.1-22.4]	27.1 [18.5-40.0]	16.9 [11.2-32.5]	14.5 [9.5-23.7]	9.9 [5.0-22.4]

Values are median [interquartile range]. Abbreviations: BMI, body-mass index; SBP, systolic blood pressure; DBP, diastolic blood pressure; NGT, normal glucose tolerance; IFG, isolated impaired fasting glucose; IGT, isolated impaired glucose tolerance; CGI, present with both IFG & IGT; New-T2DM, new type 2 diabetes; NGT-IS: insulin sensitive individuals within NGT (at the first quartile of fasting insulin within NGT); NGT-IR: insulin resistant individuals within NGT (at the top quartile of fasting insulin within NGT).

HOMA-IR = fasting glucose (mmol/L) × fasting insulin (mIU/L) / 22.5

ISI-Matsuda = 10000 / square root of [fasting glucose (mg/dL) × fasting insulin (mIU/L) × mean glucose × mean insulin concentration during OGTT]

Insulinogenic index = ($Insulin_{30\ (mIU/L)}$-$Insulin_0$)/ ($Glucose_{30(mmol/L)}$-$Glucose_0$) during OGTT.

Supplementary Figures

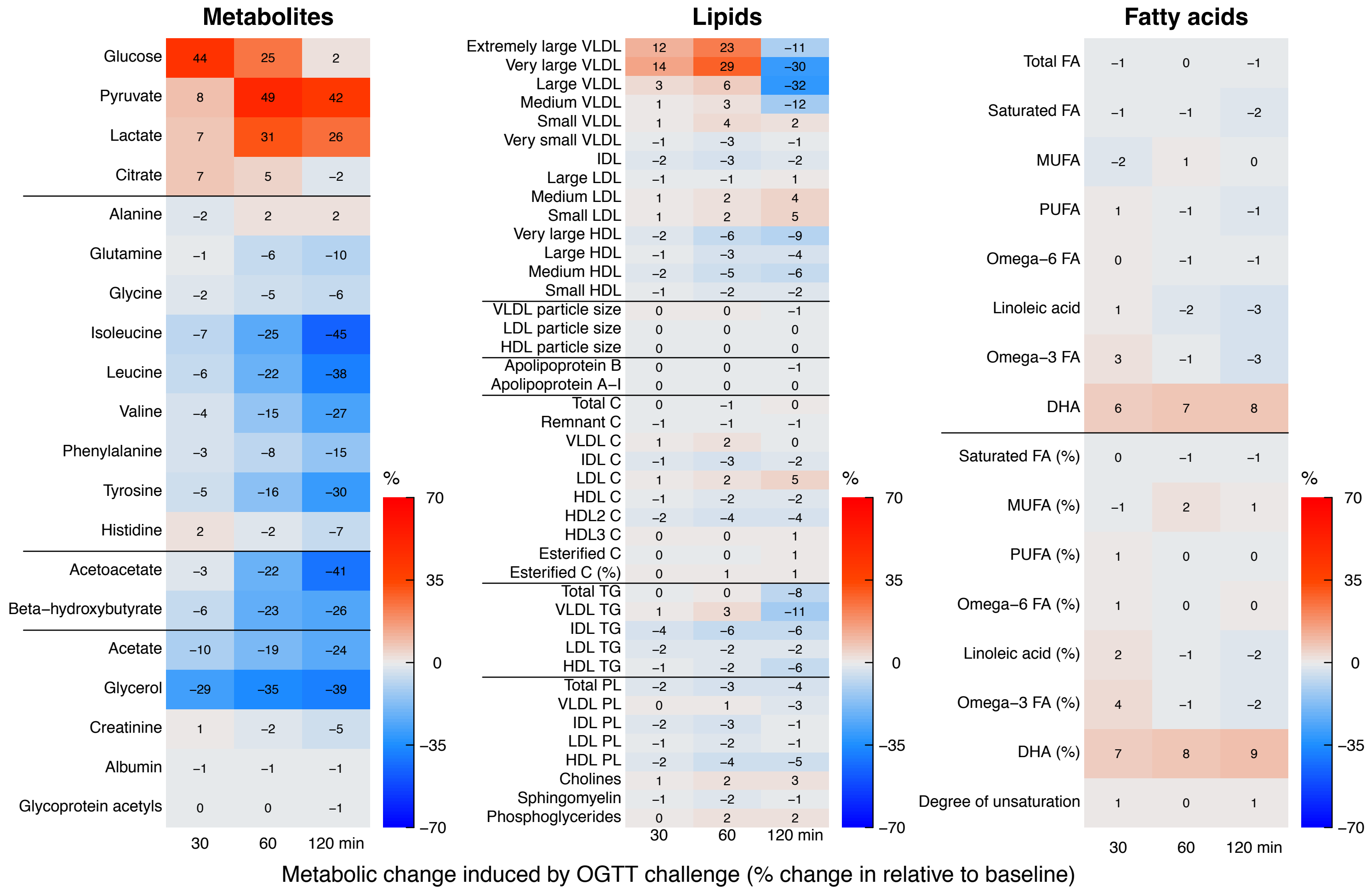

Figure S1A. Changes in 78 metabolic measures in response to an oral glucose tolerance test. The values and the colours illustrate the percent changes (metabolic changes in relative to baseline levels) at 30, 60 and 120 min for individuals with normal glucose tolerance (N=2487). Abbreviations: VLDL, very low-density lipoprotein; LDL, low-density lipoprotein; HDL, high-density lipoprotein; TG, triglycerides; PL, phospholipids; MUFA, monounsaturated fatty acids; PUFA, polyunsaturated fatty acids; DHA, docosahexaenoic acid.

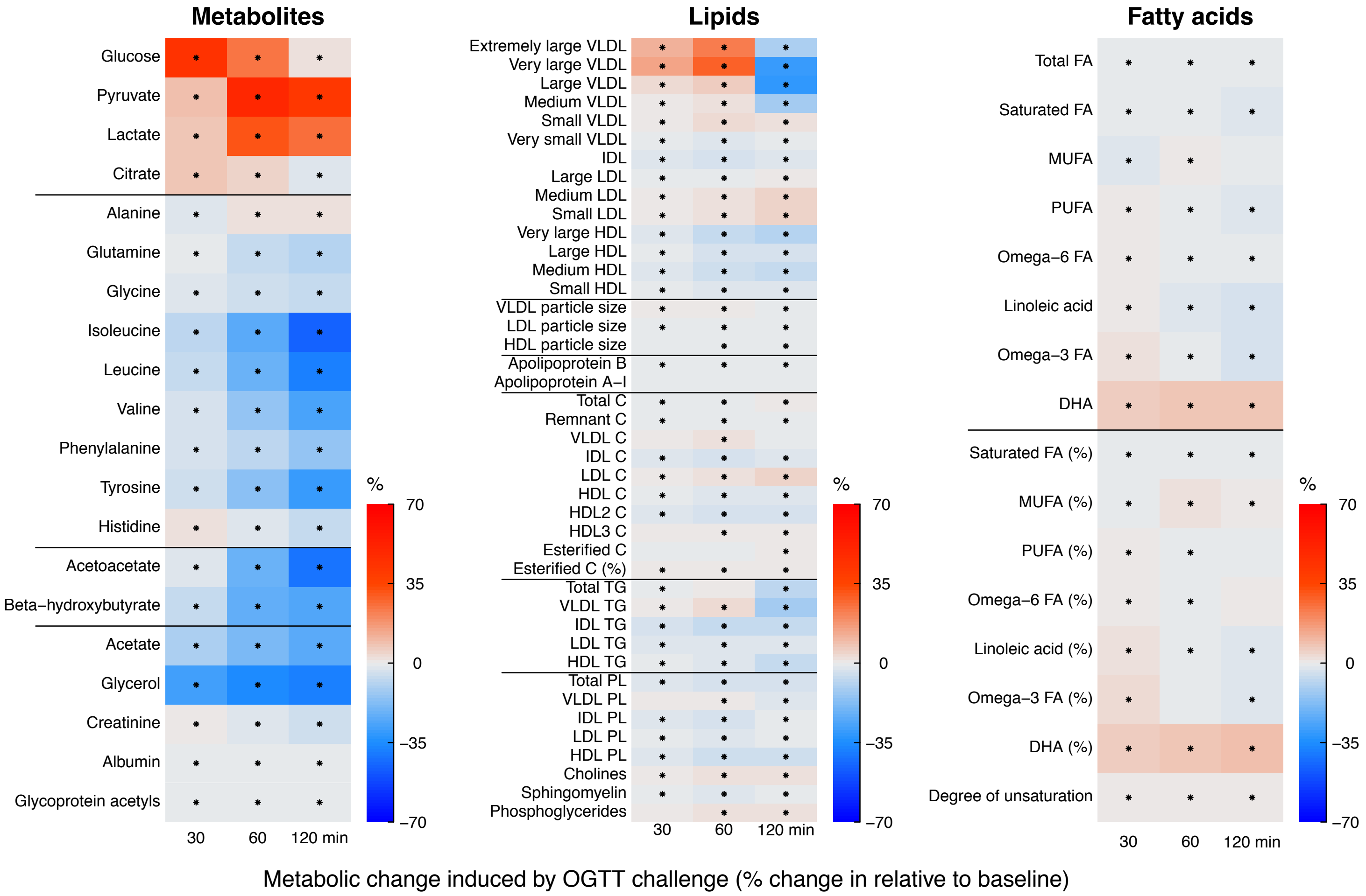

Figure S1B. Corresponding significance levels for Figure S1A. Abbreviations: VLDL, very low-density lipoprotein; LDL, low-density lipoprotein; HDL, high-density lipoprotein; TG, triglycerides; PL, phospholipids; MUFA, monounsaturated fatty acids; PUFA, polyunsaturated fatty acids; DHA, docosahexaenoic acid. *Bonferroni threshold $P < 0.0006$ for difference from baseline.

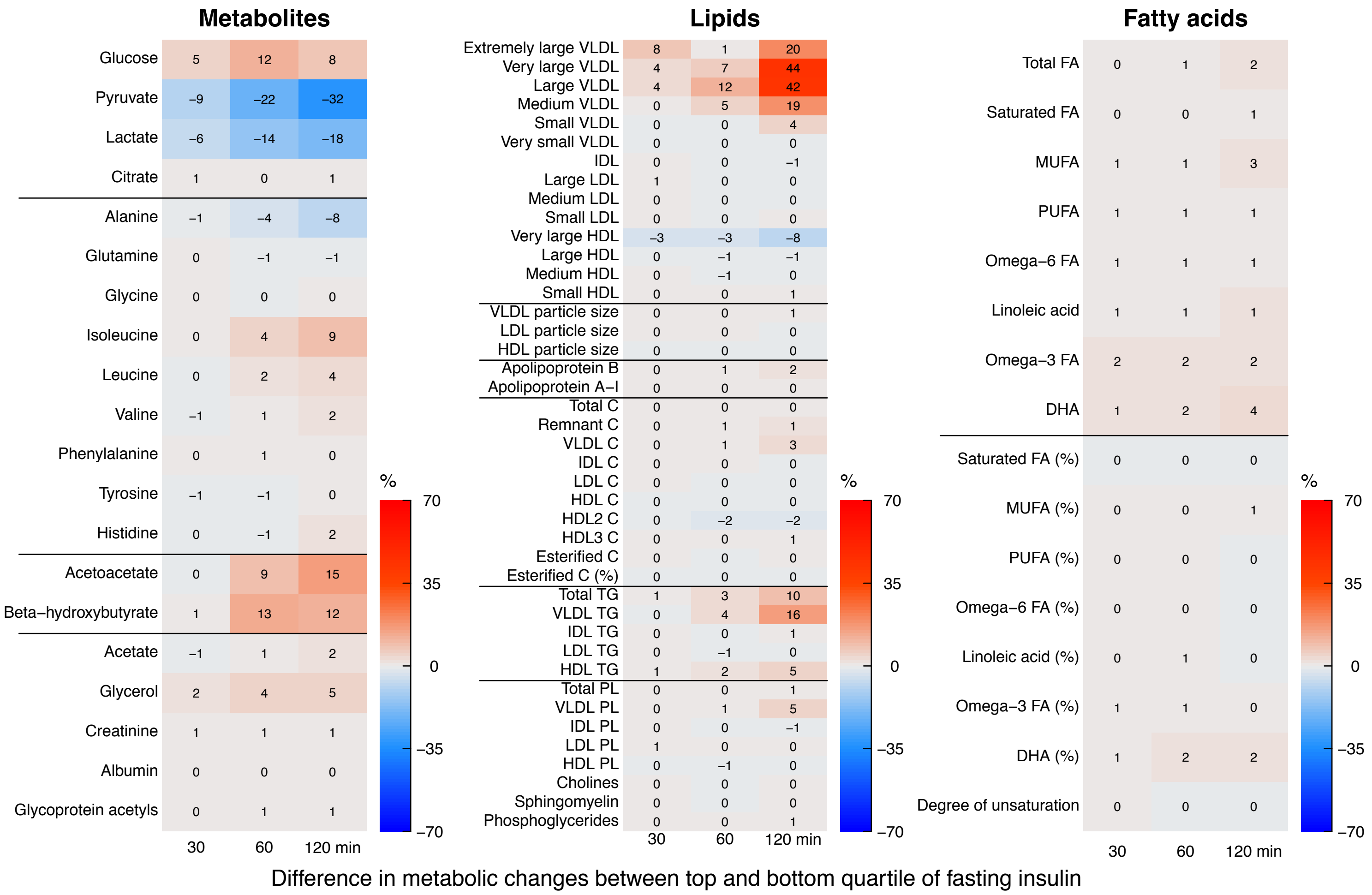

Figure S2A. Difference in metabolic changes between IR-NGT and IS-NGT. To study the effects of insulin resistance free from reverse causation, NGT individuals were further divided into insulin sensitive (IS-NGT, at the first quartile of fasting insulin within this group, n=708) and insulin resistant groups (IR-NGT, at the fourth quartile of fasting insulin, n=713). Colours and values indicated the difference in percent change between IR-NGT and IS-NGT at 30, 60 and 120 min.

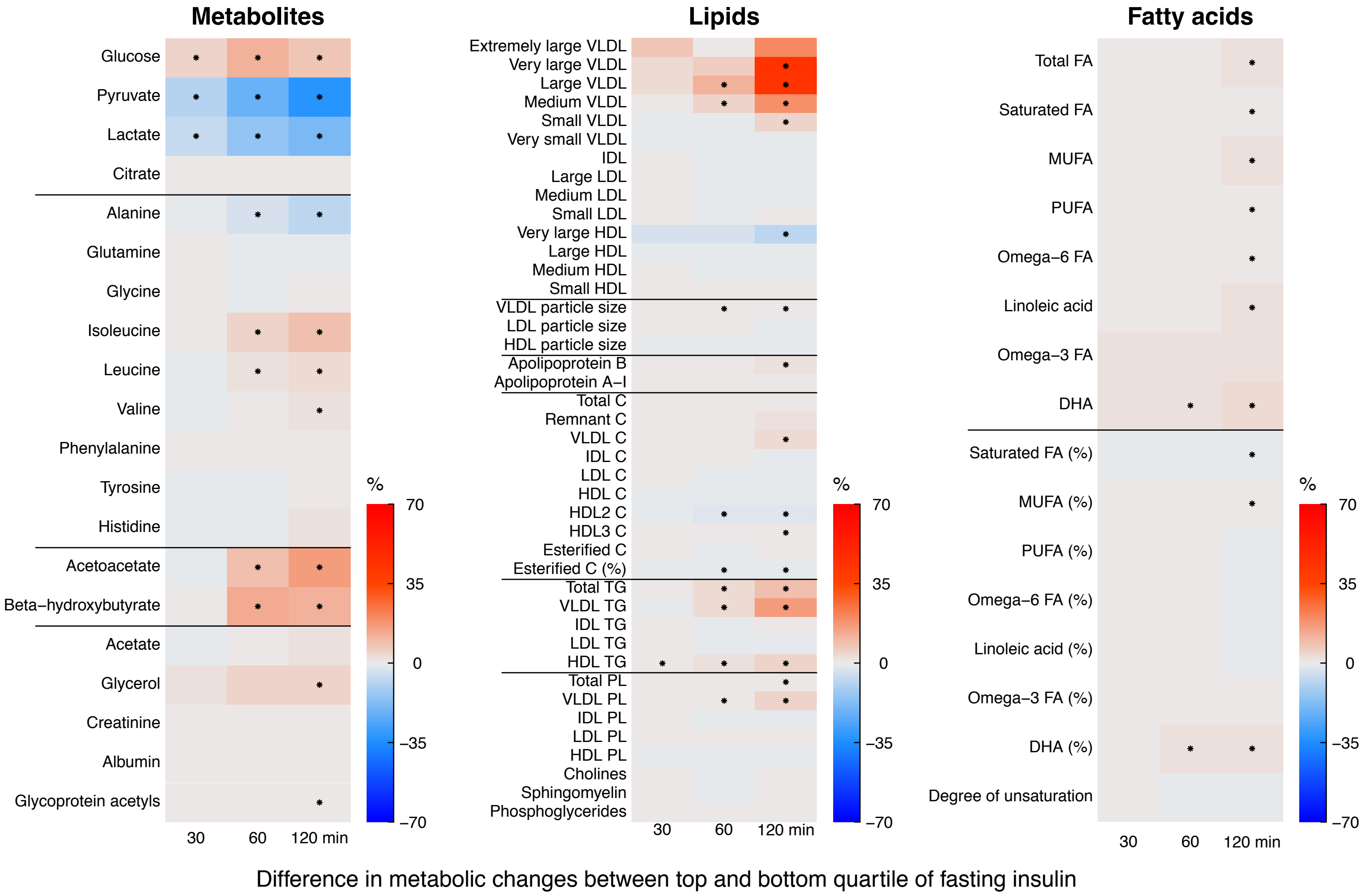

Figure S2B. Corresponding significance level for Figure S2A. *Bonferroni threshold P < 0.0006 for difference between the IS-NGT and IR-NGT groups.

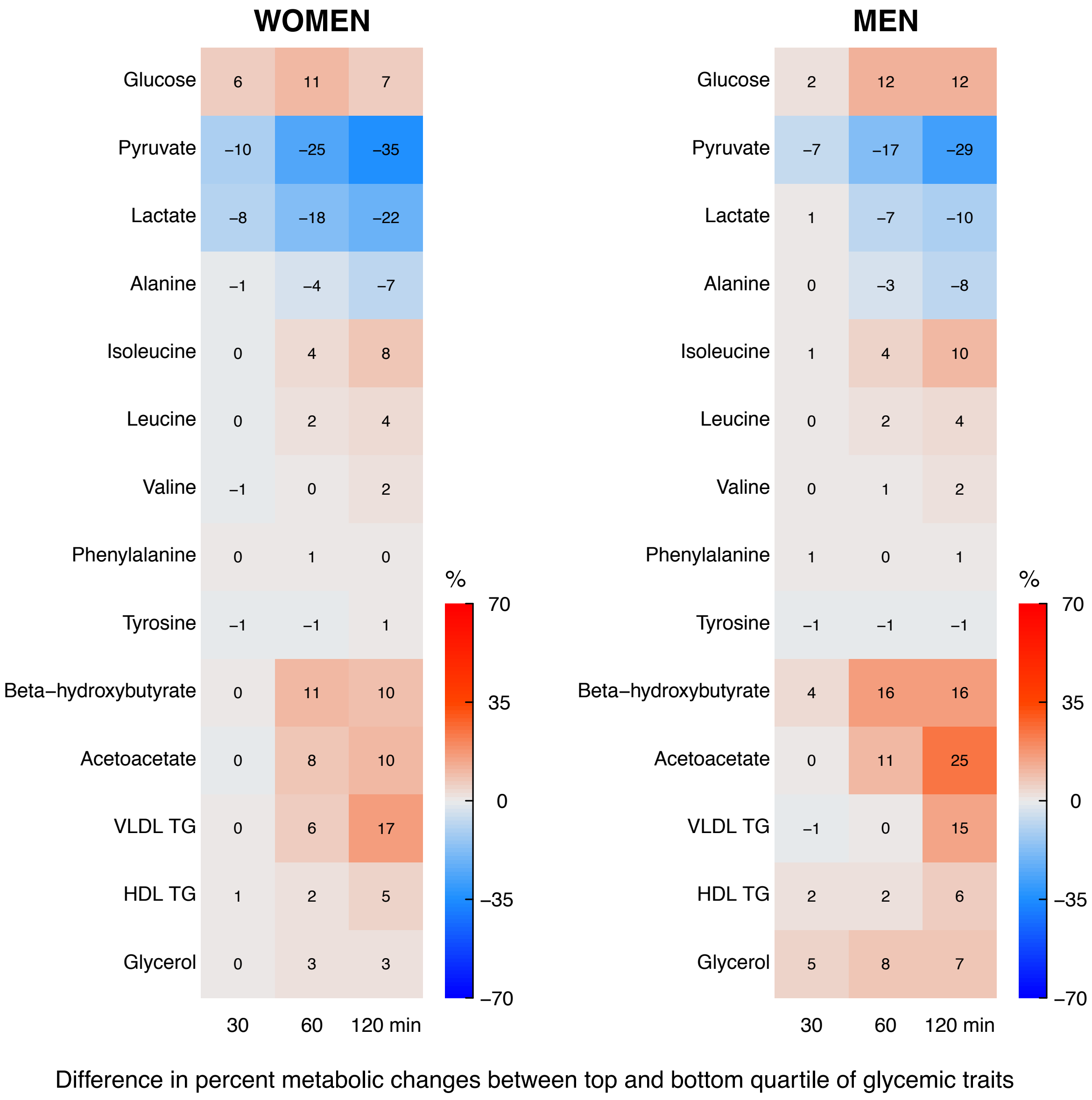

Figure S3. Differences in metabolic changes associated with insulin resistance stratified by sex. In the left plot, 472 women at the fourth quartile of fasting insulin were compared to 472 women at the first quartile of fasting insulin. In the right plot, 242 men at the fourth quartile were compared to 236 men at the first quartile. The analyses were conducted in those individuals with normal glucose tolerance.

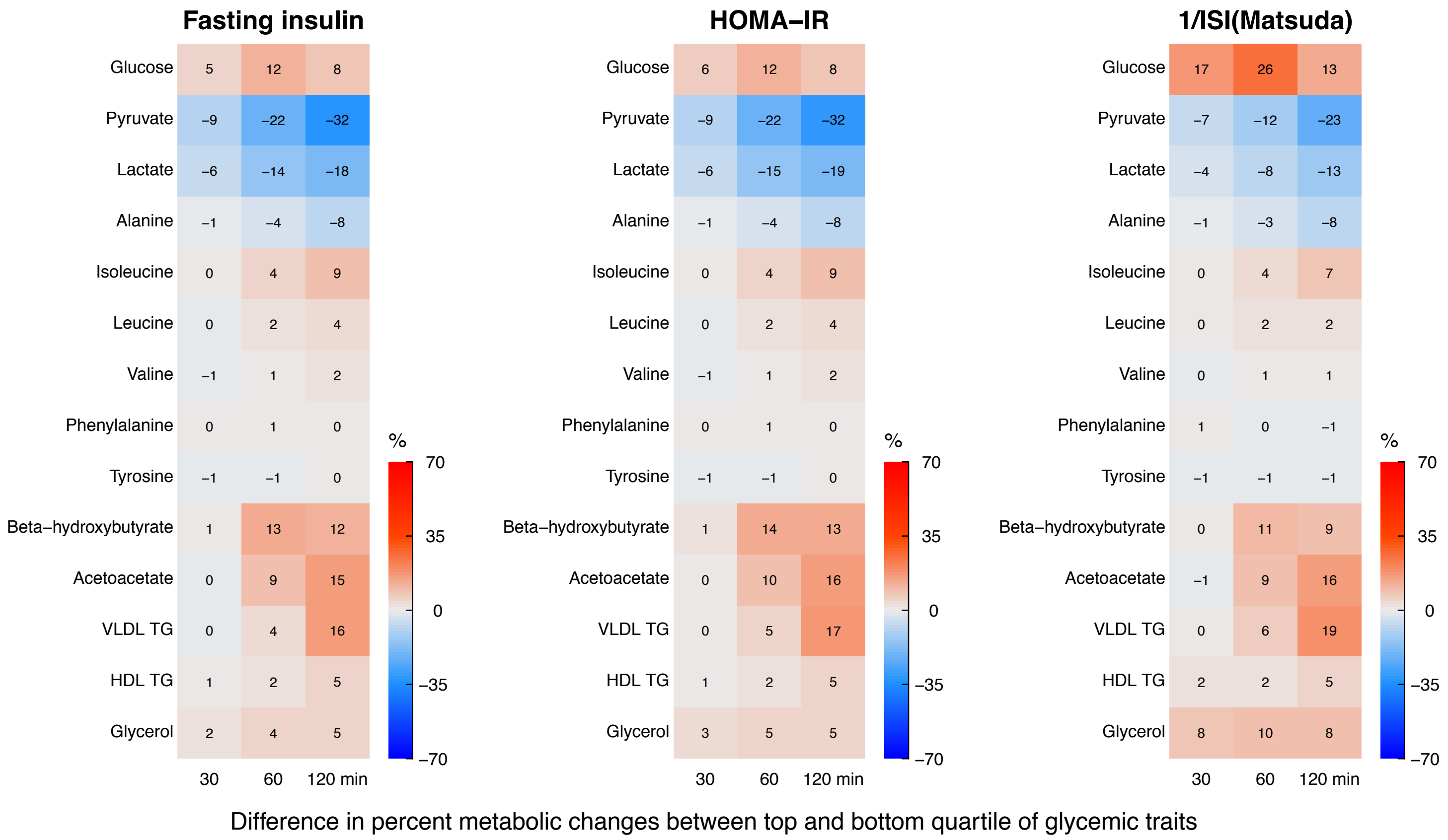

Figure S4. Differences in metabolic changes associated with fasting insulin, HOMA-IR and Matsuda-index. The metabolic changes were compared between individuals in the top and bottom quartiles of insulin resistance in NGT individuals. The insulin resistance was estimated by fasting insulin, HOMA-IR and 1/ISI(Matsuda), respectively. The results are consistent across the three indexes. In the plot, values and colours illustrate the difference in percent change between IR-NGT and IS-NGT.

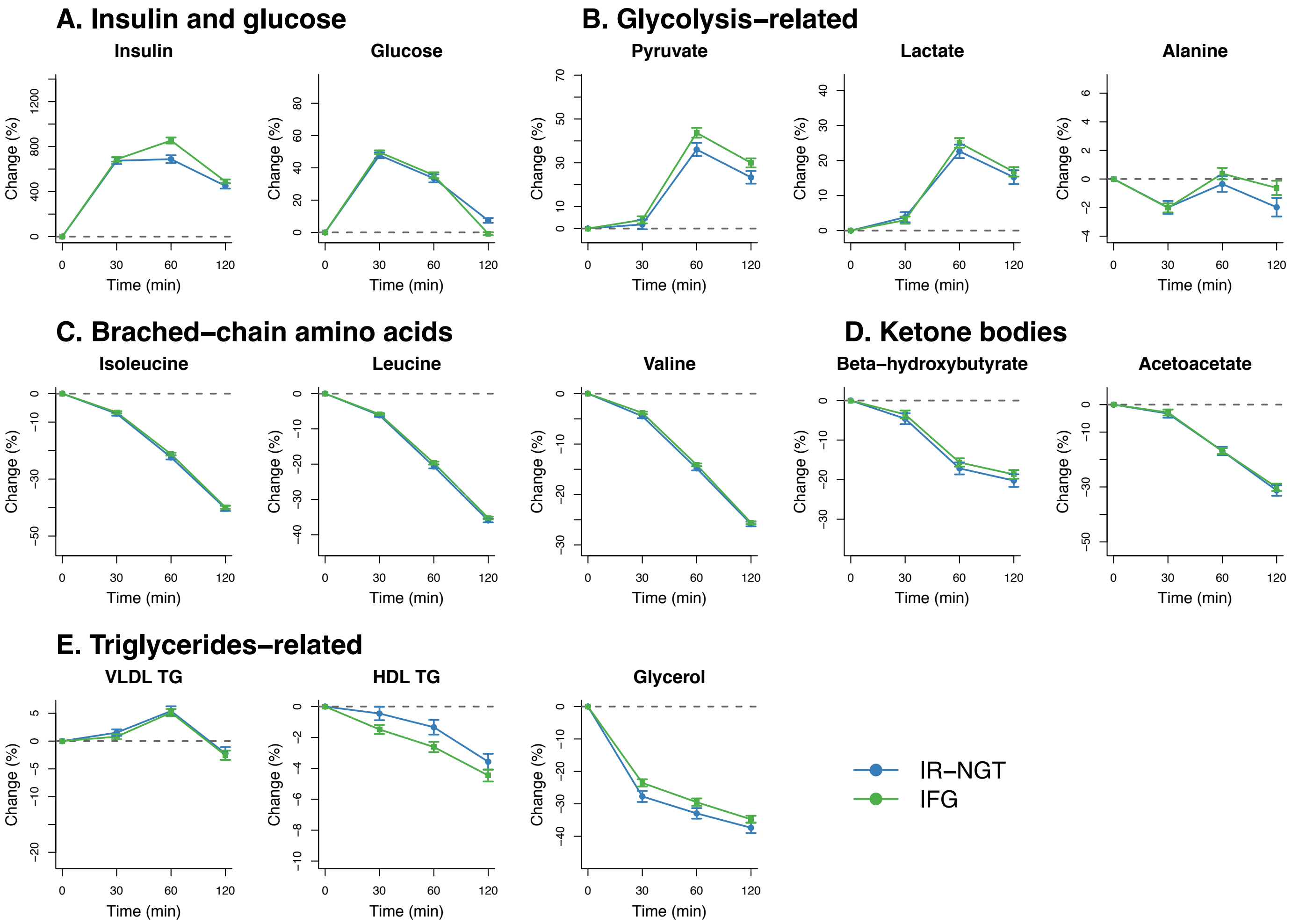

Figure S5. Metabolic trajectories compared between insulin resistant individuals of normal glucose tolerance group to those with impaired fasting glucose. IR-NGT: individuals with normal glucose tolerance and at the fourth quartile of fasting insulin within this group (n=713); IFG: Individuals with impaired fasting glucose (n=1380). The dots and error bars denote mean and 95% confidence intervals in percent change.

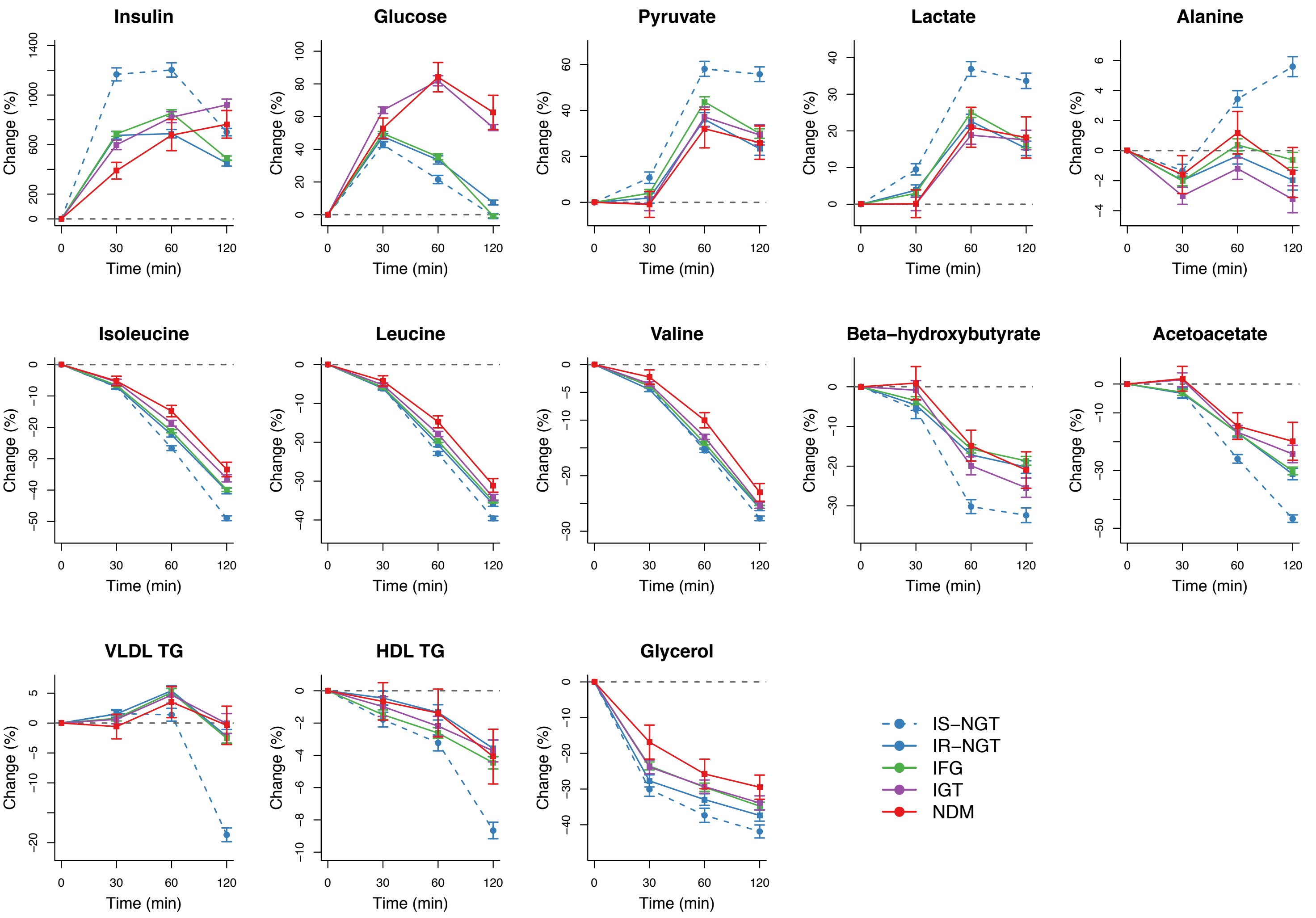

Figure S6. Metabolic trajectories for individuals with IS-NGT, IR-NGT, IFG, IGT, and NDM. Dots and error bars represent mean percent changes and 95% confidence intervals. Number of participants for IS-NGT, IR-NGT, IFG, IGT and NDM were 708, 713, 1380, 412 and 106, respectively.

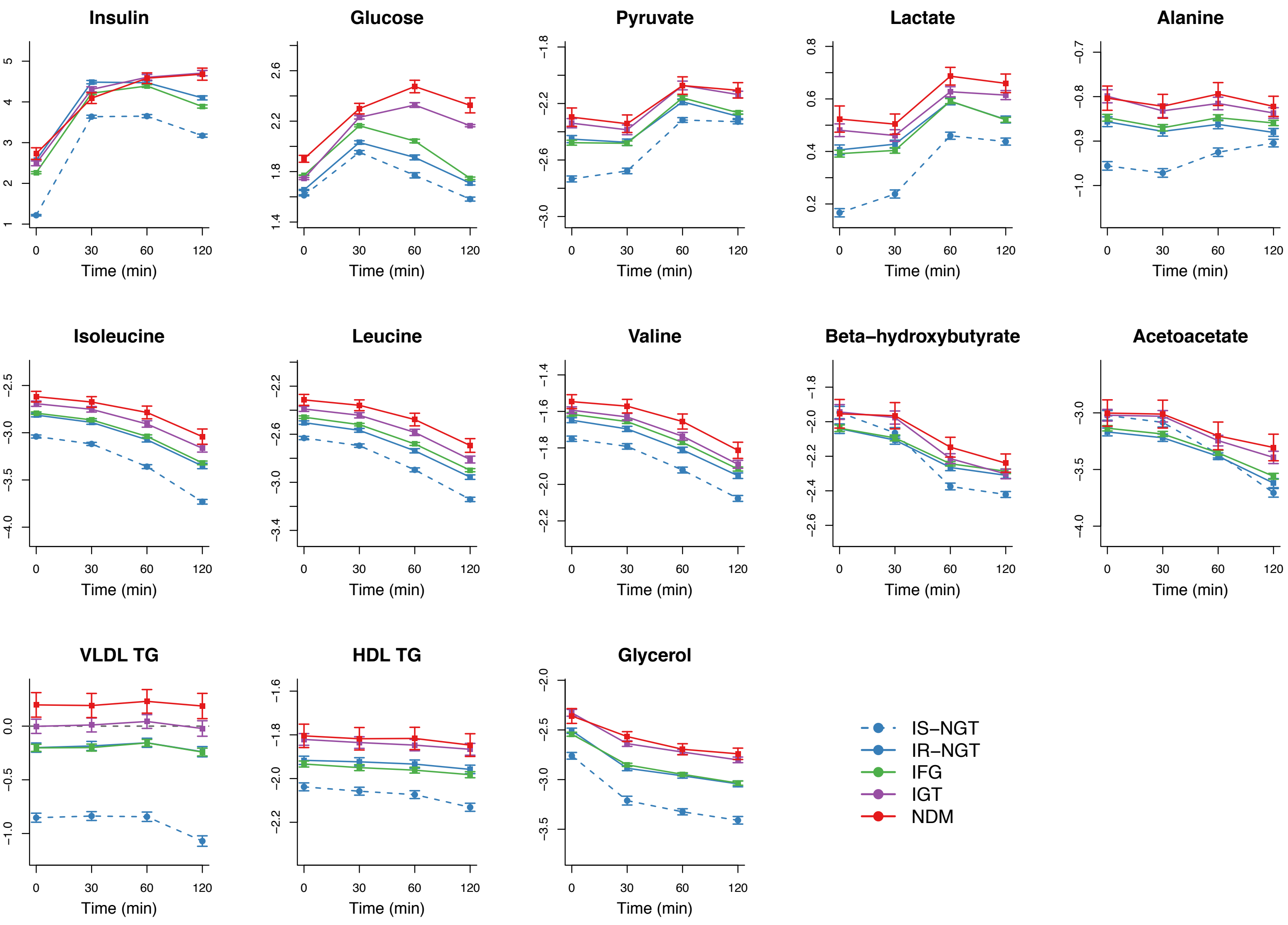

Figure S7. Absolute metabolic concentrations at 0 ,30 ,60 and 120 minute for IS-NGT, IR-NGT, IFG, IGT and NDM. The absolute concentrations were log-transformed due to skewness of the metabolic distributions. The dots and error bars indicates the mean concentration and 95% confidence intervals.

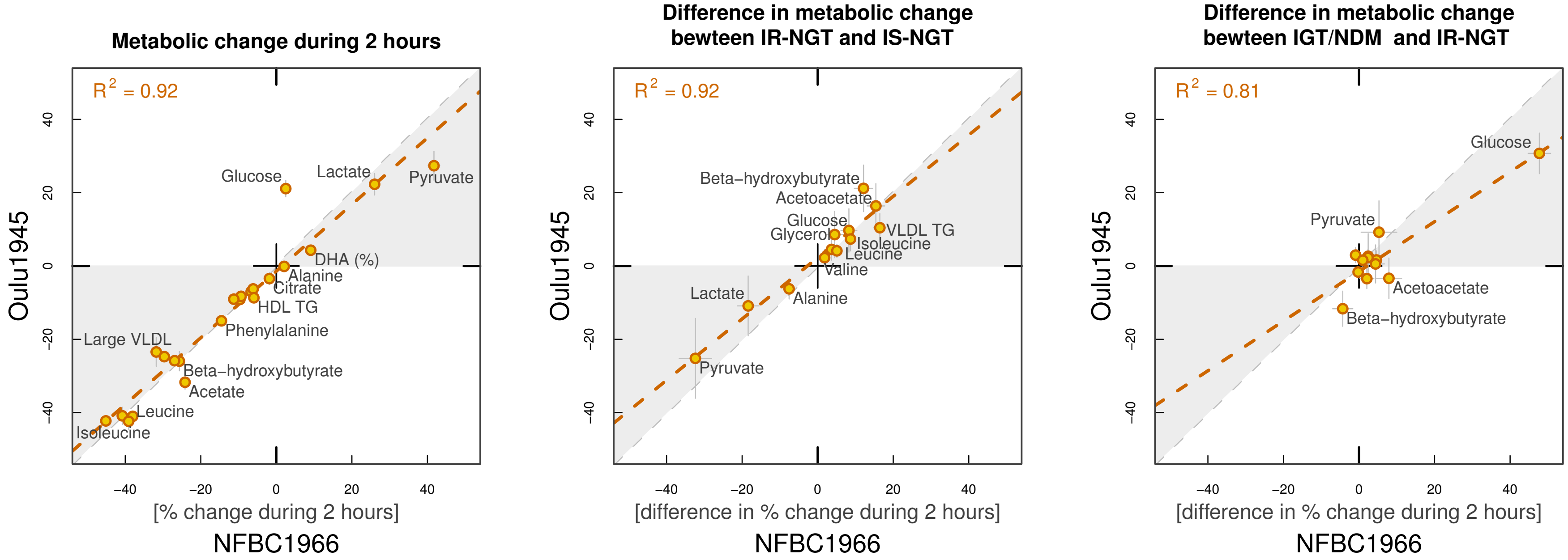

Figure S8. Replication in Oulu1945. Results discovered in NFBC66 as reported in Figure 2, 3 and 4 were replicated in a second cohort Oulu1945, corresponding to left, middle and right panel, respectively. In left panel, 22 metabolic measures were used (the same as Figure 2) and in middle and right panel, 12 measures were used (the same as Figure 3&4 except for insulin). **Left:** 2-h metabolic changes in normal glucose tolerance individuals, n=2847 and n=252 in NFBC66 and Oulu1945, respectively. **Middle:** Differences in 2-h metabolic changes comparing insulin-resistant to insulin-sensitive of NGT individuals. In NFBC66, 713 IR-NGT compared to 708 IS-NGT; In Oulu45, 64 IR-NGT compared to 62 IS-NGT. **Right:** Differences in 2-h metabolic changes comparing individuals with impaired 2-h glucose tolerance (IGT/NDM) to those insulin-resistant of NGT. In NFBC66, 518 IGT/NDM compared to 713 NGT-IR; In Oulu45, 213 IGT/NDM compared to 64 NGT-IR. Dashed lines = linear fit of the results from NFBC66 (x-axis) and Oulu45 (y-axis). R^2 = goodness of fit.

Results reported for NFBC66 as illustrated in Figure 2, 3 and 4 were further replicated in a second cohort Oulu1945, corresponding to left, middle and right panel of Figure S8 . In left panel, consistent metabolic responses in individuals with normal glucose tolerance were seen between the 2 cohorts, with 2-h metabolic changes from both cohorts falling into a straight line with an R^2 of 0.92. However, differences were noticed in 2-h glucose. Whist the middle aged NGT individuals (NFBC66) were mostly able to restore 2-h glucose back to pre-OGTT level, the older individuals (70ish, Oulu1945) still had around 20% higher glucose than baseline. In middle panel, similar differences between insulin-resistant and insulin sensitive individuals of NGT were seen for the two cohorts, with a matching result of R^2=0.92. In the right panel, both cohorts were consistently showing that although IGT or NDM individuals had higher glucose after 2 hours than insulin-resistant of NGT, the two groups otherwise had little differences in the non-glucose measures.

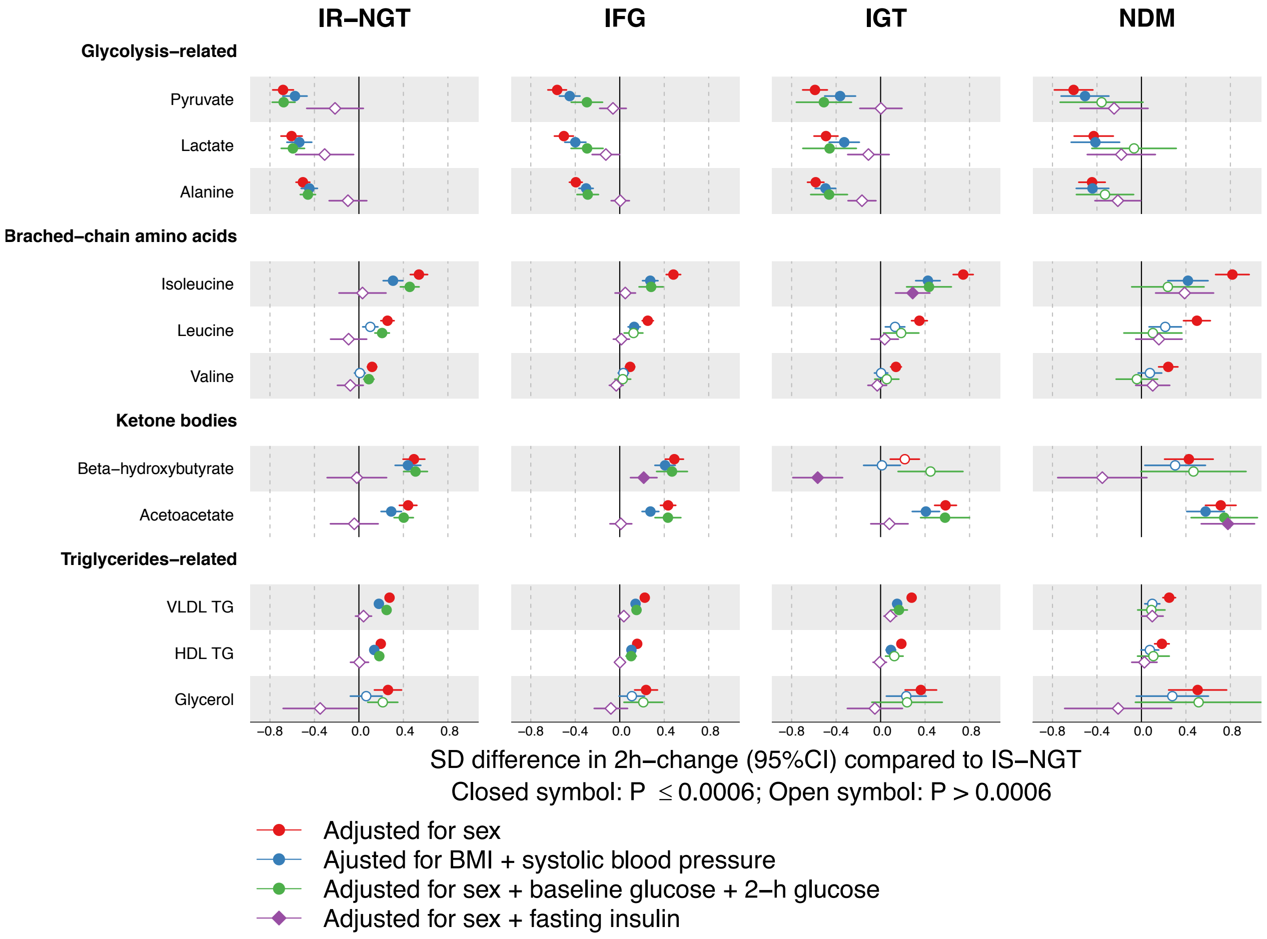

Figure S9. Group comparisons adjusted with different factors. The group comparison was analysed via linear regression model using 2-h concentration change as the response variable. Baseline and 2-h metabolite concentrations were log-transformed and the changes between 2-h and baseline metabolite concentration were scaled to baseline SD. Insulin was log-transformed. Analyses were conducted in the discovery cohort NFBC66.

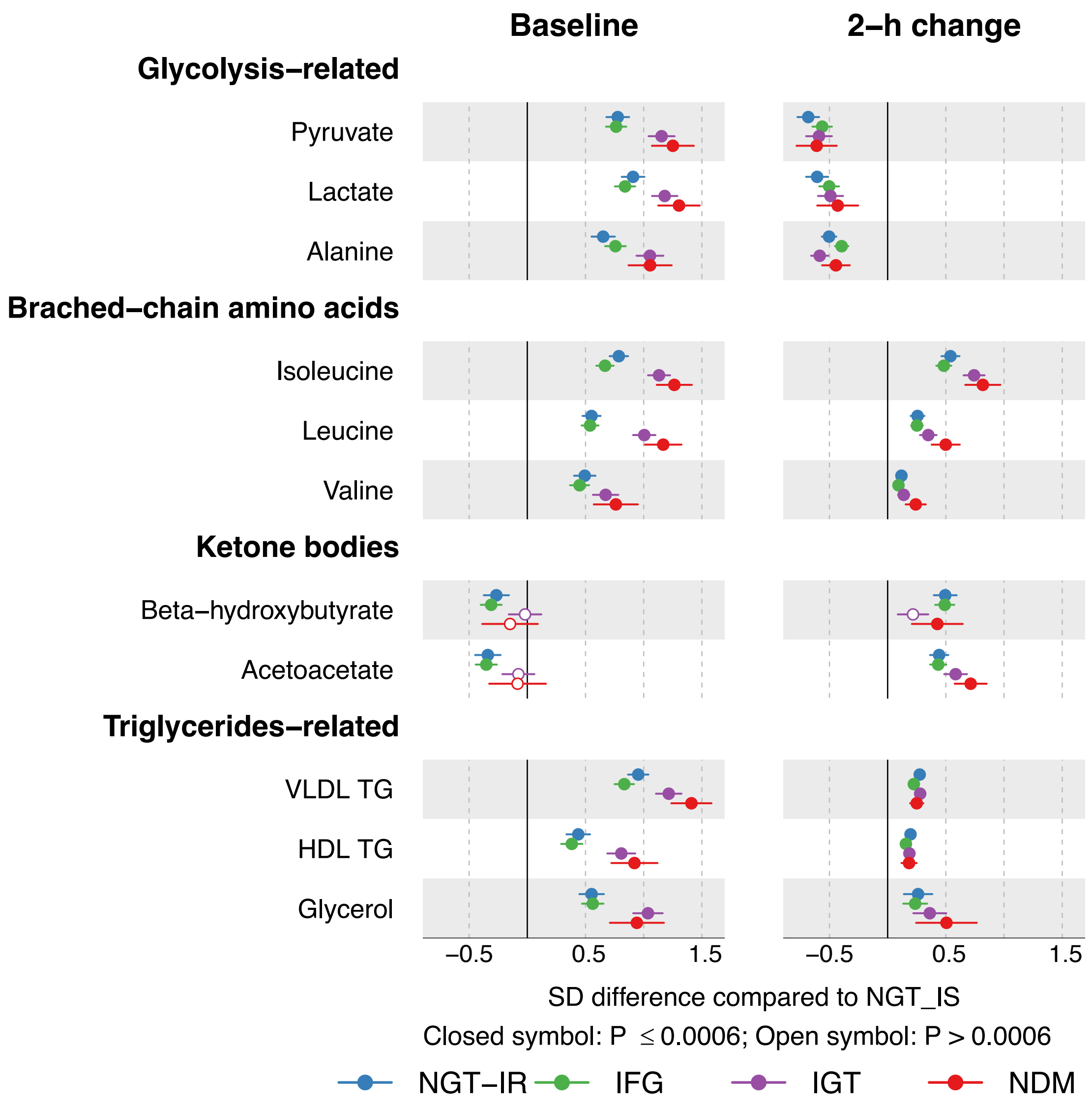

Figure S10. Difference in baseline metabolite concentration (left) and 2-h metabolic change (right) comparing the groups to the reference group (IS-NGT). Individuals with IR-NGT, IFG, IGT and NDM were compared to IS-NGT. The associations were adjusted for sex. Baseline and 2-h metabolite concentrations were log-transformed. The changes during 2 hours and baseline metabolite concentration were both scaled to baseline SD, allowing the direct comparison of the association magnitudes of the two analyses.
